# *Echinococcus granulosus sensu stricto* in Livestock and Human in Hamadan, Western Iran

**Published:** 2019

**Authors:** Mohammad MATINI, Mohammad FALLAH, Amir Hossein MAGHSOOD, Massoud SAIDIJAM, Majid FASIHI HARANDI

**Affiliations:** 1. Department of Medical Parasitology and Mycology, School of Medicine, Hamadan University of Medical Sciences, Hamadan, Iran; 2. Department of Molecular Medicine and Genetics, School of Medicine, Hamadan University of Medical Sciences, Hamadan, Iran; 3. Research Center for Hydatid Disease in Iran, Kerman University of Medical Sciences, Kerman, Iran

**Keywords:** *Echinococcus granulosus*, Genotype, Humans, Livestock, Mitochondrial genes, Iran

## Abstract

**Background:**

Cystic echinococcosis, a major public health and economic concern, is a zoonotic helminth infection with worldwide distribution. This study was conducted to investigate the genetic characteristics of hydatid cysts isolated from human and livestock in Hamadan region, western Iran during 2016–2017.

**Methods:**

Ten human hydatid cysts and 40 animal hydatid cysts including 32 sheep, 5 cattle and 3 goats were genotyped by PCR amplification of two mitochondrial genes, cox1 and nad1. Genetic identification of the isolates was performed by using bioinformatics software and mtDNA nucleotide sequences of the parasite, available in GenBank database.

**Results:**

The PCR amplification was successfully carried out on 50 hydatid cyst isolates and then the nucleotide sequencing was conducted. The sequence analysis of the samples found that the isolates belonged to *E. granulosus* sensu stricto including G1 (42/50, 84%), G2 (4/50, 8%) and G3 (4/50, 8%) genotype. The G1 genotype was detected in human (8/10, 80%), sheep (26/32, 81%), cattle (5/5, 100%) and goat (3/3, 100%) hydatid cysts. The G2 and G3 genotypes were found only in sheep and human isolates. Alignment analysis of the cox1 and nad1 gene sequences revealed thirteen and ten sequence types, respectively.

**Conclusion:**

G1 was the prevailing genotype of *E. granulosus* in the area and dog-sheep transmission cycle should be considered when implementing hydatidosis control programs. In addition, high genetic diversity was detected among the hydatid cyst isolates.

## Introduction

*H*ydatidosis, a global parasitic zoonosis, is caused by the larval stage of *Echinococcus granulosus* sensu lato classified as a genus of small tapeworms in the Taeniidae family. In the life cycle of the parasite, herbivores and canids act as the intermediate and definitive hosts, respectively and principally, livestock and dogs are responsible for the spread of the infection worldwide. The hydatid cyst or the metacestode stage of the parasite is gradually formed in inner organs of the intermediate host after ingestion of the parasite eggs (1). Liver and lung are the most common involved organs with hydatid cyst in up to 65% and 25% of cases, respectively (2). Moreover, a human, as an accidental intermediate host, can be infected by the oral route (1). Echinococcosis/hydatidosis is a significant public health problem and economic concern in most parts of the world, especially in highly endemic areas such as Australia, Northern and Eastern Africa, Southern America, the Middle East, Western and Central Asia, and China (2, 3). Iran is one of the endemic areas of echinococcosis with a considerable prevalence of 5%–49% among stray dogs and a mean of 6.73% in livestock (4).

Taxonomy of *Echinococcus* species has been controversial for a long time but recently, phylogenetic analysis of *E. granulosus* s.l. has shown that the current classification needs to be revised. Molecular characterization of *E. granulosus* s.l. based on both mitochondrial and nuclear rRNA genes revealed that this species contains at least four distinct species, including *E. granulosus* s.s (G1–G3 complex), *E. equinus* (G4), *E.* *ortleppi* (G5) and *E. canadensis* (G6–G10) (5–7). Although the situation of *E. canadensis* is uncertain and it could be divided into two species; *E. canadensis* (G8 and G10) and *E. intermedius* (G6/G7) (3, 8). The genotypic variations are emerged in phenotypic characteristics and can affect biological behavior of the parasite such as host specificity, life cycle pattern, pathogenicity, treatment, transmission dynamics, epidemiology and consequently, ways to control and prevention it (9).

Phylogenetic analysis and genetic characterization of the parasite have been shown that *E. granulosus* s.s and camel genotype are the most species of *E. granulosus* in Iran (10–14). Hamadan Province, located in west of Iran, is one of the endemic areas of echinococcosis (15,16) Due to little information about genetic characterization of the parasites in the region, this study was conducted, based on mitochondrial genes (cox1 and nad1) analysis.

## Materials and Methods

### Parasites

Forty livestock hydatid cysts including sheep (thirty-two samples), cattle (five samples), goats (three samples), and ten human hydatid cysts were collected periodically from Hamadan industrial slaughterhouse and Beast Hospital of Hamadan City, respectively, during 2016–2017. Hydatid cysts with transparent fluid and whitish germinal layer those were fertile selected for molecular testing. Protoscoleces from individual hydatid cyst were aspirated under aseptic condition by a sterile needle. Then, they were washed three times with sterile normal saline solution and conserved in 70% (v/v) ethanol at −20 °C, until use for molecular examination.

The study was approved by Ethics Committee of the university.

### DNA extraction and PCR amplification

After removal of the ethanol and washing of the protoscolices with sterile distilled water, genomic DNA extraction was carried out on approximately 50 mg protoscolices pellet of each individual hydatid cyst by using High Pure PCR Template Preparation Kit (Roche Diagnostics, Mannheim, Germany) based on manufacturer’s instructions. Quality of the extracted DNA was assessed by spectrophotometer (NanoDrop-ND1000) and the DNA preserved at −20 °C until PCR analysis.

Two partial sequences of mitochondrial genes of *E. granulosus* s.l. were amplified separately from individual DNA isolates by using primer sets JB3/JB4.5 and JB11/JB12 for amplification of cox1 and nad1 genes, respectively (17, 18).

PCR reaction was carried out in a final volume of 50 μl, including 5 μl gDNA (50–100 ng), 25 pmol of each primer, 5 μl buffer 10X, 250 μM of each dNTP’s, 3.5 mM MgCl2 (25mM), 2 units Taq DNA polymerase (Sinagene Company) and sterile distilled water up to the final volume. Positive control and negative control (without any DNA) were included in each PCR run. The PCR profile was as follows: primary denaturation at 95 °C for 3 min, followed by 35 cycles of denaturation (30 sec at 94 °C), annealing (45 sec at 50 °C), extension (35 sec at 72 °C), and then final extension at 72 °C for 10 min. After amplification, the PCR product was evaluated by electrophoresis in a 1.5% (w/v) Tris-borate/EDTA (TBE) agarose gels stained with SYBR Safe DNA gel stain (Invitrogen).

### Sequencing and phylogenetic analysis

The PCR amplicons of cox1 and nad1 genes were purified and were subjected to sequencing by using Applied Biosystems Automated 3730xl DNA Analyzer (Bioneer Inc., Korea) and the same primers utilized in the primary amplification. The electropherogram of sequences was checked visually and sequence editing and alignment of the sequences were done by Chromas software, version 2.6, and BioEdit software, version 7.2.

Sequence identity of the consensus sequences of cox1 and nad1 genes and comparison with GenBank reference sequences were performed using the BLASTn program (https://blast.ncbi.nlm.nih.gov/Blast.cgi?PAGE_TYPE=BlastSearch). Analysis of single nucleotide polymorphisms (SNPs) was conducted using DnaSP V5 software.

Phylogenetic analysis of nucleotide sequences was done by using the consensus sequences of cox1 (Hamc1-Hamc13) and nad1 (Hamn1-Hamn10) genes in this study that they had represented nucleotide variations and reference sequences of *E. granulosus* genotypes (G1–G10) and *Taenia saginata*, as outgroup.

*Maximum likelihood* methods and bootstrap analysis were applied for construction of phylogenic trees and evaluation of the reliability of the inferred trees, respectively, using MEGA software version 6.

## Results

Out of 40 animal hydatid cyst isolates 24 (60%) belonged to lung and 16 (40%) to liver and 10 human hydatid cyst samples included five (50%) lung cysts, four (40%) liver cysts and one (10%) peritoneal cyst. PCR amplification of the cox1 and nad1 genes was successfully carried out on the DNA extracted samples and yielded amplicons of approximately 450 and 500 bp in length, respectively. In each PCR run, no amplification was detected in negative control. The amplicons of the mitochondrial genes were subjected to nucleotide sequencing and yielded consensus sequences consisting of 394 bp for cox1 gene and 471 bp for nad1 gene. Thirteen and ten different sequence types were detected by alignment of the sequences of cox1 and nad1 genes, respectively. Sequence types of the two mitochondrial genes, related to the isolates, are available in GenBank with accession numbers MG792551-73 (Tables 1 and 2).

**Table 1:** Multiple alignments of the cox1 gene sequences of *Echinococcus granulosus* isolated in Hamadan and reference sequences (genotype G1, G2 and G3)

***Haplotypes***	***Accession number***	***Number (%)***	***Nucleotide position of variable sites^a^***										
cox1			38	56	66	111	174	204	228	231	238	257	312
G1	U50464	---	T	C	C	C	C	T	A	T	T	T	G
Hamc1	MG792551	18 (36)	.	.	.	.	.	.	.	.	.	.	.
Hamc2	MG792552	9 (18)	.	T	.	.	.	.	.	.	.	.	.
Hamc3	MG792553	3 (6)	.	.	.	t	.	.	.	.	.	.	.
Hamc4	MG792554	3 (6)	.	.	.	.	.	.	g	.	.	.	.
Hamc5	MG792555	2 (4)	.	.	.	.	.	.	.	c	.	.	.
Hamc6	MG792556	1 (2)	.	.	.	.	.	.	.	.	.	.	t
Hamc7	MG792557	1 (2)	.	.	.	.	.	a	.	.	.	.	.
Hamc8	MG792558	1 (2)	C	T	.	.	.	.	.	.	.	.	.
Hamc9	MG792559	2 (4)	.	T	.	.	.	.	.	.	c	.	.
Hamc13	MG792563	2 (4)	.	.	t	.	.	.	.	.	.	.	.
G2	M84662	---	.	T	t	.	.	.	.	.	.	C	.
Hamc10	MG792560	4 (8)	.	T	t	.	.	.	.	.	.	C	.
G3	M84663	---	.	.	t	.	.	.	.	.	.	C	.
Hamc11	MG792561	1 (2)	.	.	t	.	.	.	.	.	.	C	.
Hamc12	MG792562	3 (6)	.	.	t	.	t	.	.	.	.	C	.

a Dots indicate identity with reference sequences. Non-synonymous substitutions are indicated in capital letters and synonymous substitutions are in lower cases

**Table 2:** Multiple alignments of the nad1 gene sequences of *Echinococcus granulosus* isolated in Hamadan and reference sequences (genotype G1, G2 and G3)

***Haplotypes***	***Accession number***	***Number (%)***	***Nucleotide position of variable sites^a^***										
nad1			40	43	57	150	151	243	282	343	356	375	429
G1	AJ237632	---	G	A	T	T	T	T	C	A	A	T	A
Hamn1	MG792564	7 (14)	.	.	.	c	.	.	t	.	.	.	.
Hamn2	MG792565	19 (38)	.	.	.	.	.	.	t	.	.	.	.
Hamn3	MG792566	1 (2)	.	.	.	.	.	.	t	.	G	.	.
Hamn4	MG792567	2 (4)	A	.	.	.	.	.	t	.	.	.	.
Hamn5	MG792568	1 (2)	.	.	.	.	.	c	t	G	.	.	.
Hamn6	MG792569	1 (2)	.	.	c	c	.	.	t	.	.	.	.
Hamn7	MG792570	1 (2)	.	.	.	.	C	.	.	.	.	.	.
Hamn8	MG792571	3 (6)	.	.	.	.	.	.	.	.	.	.	.
Hamn9	MG792572	7 (14)	.	.	.	.	.	.	t	.	.	c	.
G2	AJ237633	---	.	G	.	.	.	.	t	.	.	.	G
G3	AJ237634	---	.	G	.	.	.	.	t	.	.	.	G
Hamn10	MG792573	8 (16)	.	G	.	.	.	.	t	.	.	.	G

a Dots indicate identity with reference sequences. Non-synonymous substitutions are indicated in capital letters and synonymous substitutions are in lower cases

BLAST analysis of the cox1 and nad1 sequences, using GenBank sequences, revealed that the isolates belonged to *E. granulosus* s.s including G1 (common sheep strain, 84%; 42/50), G2 (Tasmanian sheep strain, 8%; 4/50) and G3 genotype (buffalo strain, 8%; 4/50). Among the human hydatid cysts, eight isolates belonged to G1 and one isolate identified as G2 and another was G3 genotype. In livestock samples, the G2 and the G3 genotypes were detected only in the sheep hydatid cysts.

The isolates with G1 genotype had ten cox1 sequence types and nine nad1 sequence types and also, the isolates with G2 and G3 genotype had one and two cox1 sequence types, respectively (Tables 1 and 2). In this study, one nad1 sequence type was detected for G2/G3 genotype.

**Fig. 1:**
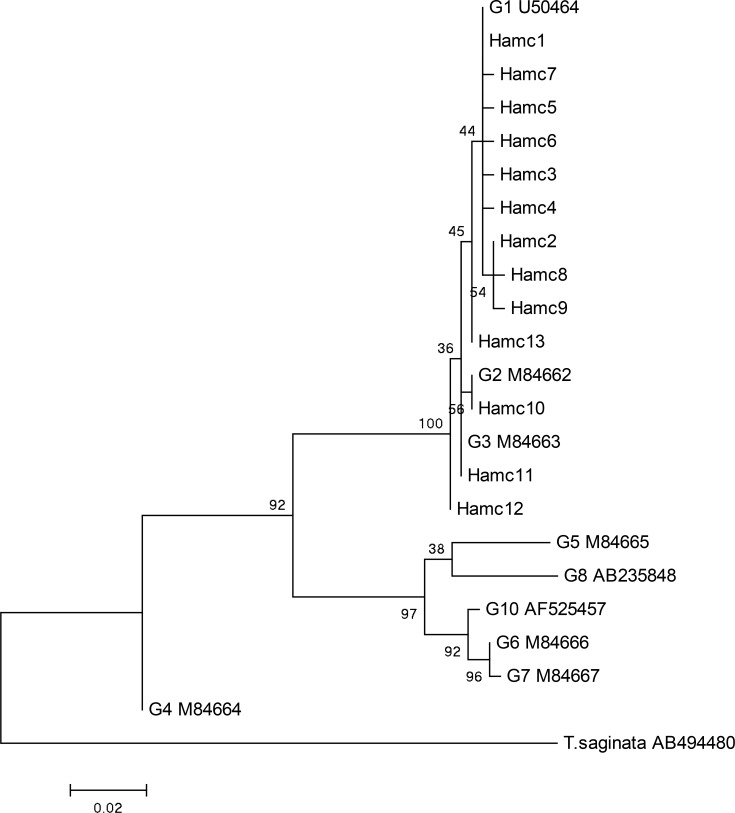
Genetic relationships of *Echinococcus granulosus* isolates from Hamadan and reference sequences including genotypes of *E. granulosus* and *Taenia saginata* as outgroup. The phylogenetic analysis was conducted using cox1 gene and the maximum likelihood method based on the Tamura-Nei model in MEGA 6

**Fig. 2:**
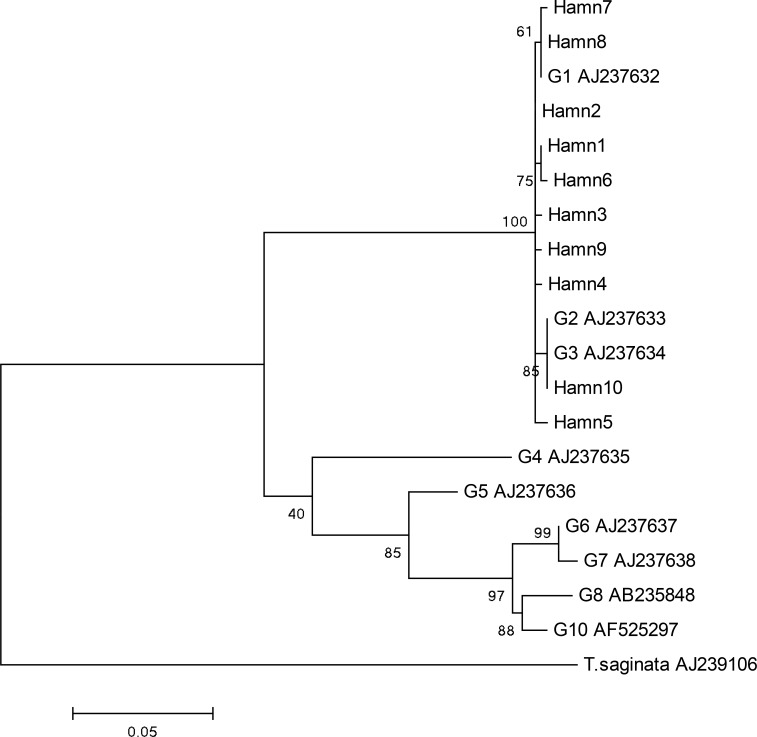
Genetic relationships of *Echinococcus granulosus* isolates from Hamadan and reference sequences including genotypes of *E. granulosus* and *Taenia saginata* as outgroup. The phylogenetic analysis was conducted using nad1 gene and the maximum likelihood method based on the Tamura-Nei model in MEGA 6

The analysis of cox1 and nad1 genes showed some nucleotide substitutions with no insertions/deletions. Eleven variable sites were found in each of the two analysed regions including eight singleton variable sites and three parsimony informative sites (positions: 56, 66, 257) in the cox1 region, and seven singleton variable sites and four parsimony informative sites (positions: 43, 150, 282, 429) in the nad1 region. Of these 22 point mutation sites, three and six non-synonymous substitutions were found in the cox1 and nad1 regions, respectively (Tables 1 and 2). Two phylogenetic trees based on cox1 (Fig. 1) and nad1 (Fig. 2) regions were constructed to show genetic relationships between the isolates and other genotypes of *E. granulosus*, available in GenBank database. The phylogenetic analysis revealed the separate clade consists of the isolates and the G1, G2 and G3 reference genotypes (Figs. 1, 2).

## Discussion

In endemic regions of hydatidosis, molecular epidemiology and genetic characterization of *E. granulosus* is needed to implement control programs of the disease because genetic variations of the parasite may affect the life cycle pattern, host specificity, transmission dynamics, pathogenicity and other epidemiological aspects of *E. granulosus* (9).

Previous epidemiological studies have indicated that some genotypes/strains of the parasite, consist of *E. granulosus* sensu stricto complex (G1, G2 and G3 genotypes) and camel strain (G6 genotype) are present in Iran based on mitochondrial and nuclear rRNA genes analysis (10–14). However, recently one camel hydatid cyst was identified as genotype G5 *(E. ortleppi*) in Isfahan Province, central part of Iran (19) and six goat hydatid cysts were diagnosed as genotype G7 (related to *E. canadensis*) in north-eastern Iran (20).

In the present study, three genotypes of *E. granulosus* (G1, G2 and G3) were detected in Hamadan region, based on mitochondrial genes analysis, and G1 was the most prevalent genotypes in this area (84%). This finding is consistent with the results of some studies conducted in other parts of Iran (13, 21, 22). Common sheep strain (G1) was the only genotype of the parasite detected in human and livestock hydatid cysts (23,24).

G6 is another less common genotype in Iran (10–14) not detected in this study. This finding can be justified by the lack of camel breeding, besides of not commonly used camel meat in the area. The sheep–dog cycle is actively involved in the transmission of cystic echinococcosis in this region.

In the present study, analysis of cox1 gene yielded thirteen different sequence types or haplotypes. Hamc1 haplotype was the most sequence type (36%) whose nucleotide sequence was 100% similar to the sequence of the reference sequence (25), and a number of sequences available in GenBank (KX87471, KY499577 and MF00429). The Hamc7 and Hamc9 sequence types were at most 99% similar to the sequence types reported from northern Iraq (Kurdistan region) (26) and Iran (27), respectively, considered as new haplotypes. The other sequence types detected in this study had 100% similarity with those reported from Iran and other parts of the world.

Moreover, in this study, sequences alignment analysis of the nad1 showed ten distinct sequence types. The Hamn2 haplotype was the most prevalent (38%) sequence type and had 100% similarity with some sequences reported from Iran (KJ162552 and KJ162553) and other parts of the world (KY881715, KX039965, and KU925431). Maximum nucleotide sequence homology of the Hamn3-Hamn7 haplotypes with other sequences in GenBank was 99% and the haplotypes were reported for the first time. The Hamn10 sequence type that identified as G2/G3 genotype had 100% similarity with some sequences available in GenBank such as the sequence previously reported from Iran (GQ357999) (28) and the reference sequences (AJ237633/AJ237634) (18) and also it had 99% homology with the sequence reported from Iranian camel hydatid cyst (27).

## Conclusion

This common *E. granulosus* genotypes in sheep, cattle and goat reported from other parts of Iran, were also present in Hamadan province, western Iran. In addition, high genetic diversity was observed among *E. granulosus* s.s isolates and genotype G1 was the prevailing genotype of the parasite in this region that can affect human and animals. Therefore, the potential of infected livestock should be considered as an important reservoir for human hydatidosis.
